# Danish guidelines for management of non-*APC*-associated hereditary polyposis syndromes

**DOI:** 10.1186/s13053-021-00197-8

**Published:** 2021-10-07

**Authors:** Anne Marie Jelsig, John Gásdal Karstensen, Niels Jespersen, Zohreh Ketabi, Charlotte Lautrup, Karina Rønlund, Lone Sunde, Karin Wadt, Ole Thorlacius-Ussing, Niels Qvist

**Affiliations:** 1 Department of Clinical Genetics, University Hospital of Copenhagen, Rigshospitalet, Copenhagen, Denmark; 2 Danish Polyposis Registry, Gastrounit, Hvidovre Hospital, Hvidovre, Denmark; 3 Department of Clinical Medicine, University of Copenhagen, Copenhagen, Denmark; 4 Department of Gynecology and Obstetrics, University Hospital of Copenhagen, Rigshospitalet, Copenhagen, Denmark; 5 Department of Clinical Genetics, Aalborg University Hospital, Aalborg, Denmark; 6 Department of Clinical Genetics, University Hospital of Southern Denmark, Vejle Hospital, Vejle, Denmark; 7 Department of Gastrointestinal Surgery, Aalborg University Hospital, Aalborg, Denmark; 8 Department of Clinical Medicine, Aalborg University, Aalborg, Denmark; 9 Research Unit for Surgery, Odense University Hospital, Odense, Denmark; 10 University of Southern Denmark, Odense, Denmark

**Keywords:** Cancer, Polyposis, Genetics, Hereditary, Surveillance, Management, Guideline

## Abstract

Hereditary Polyposis Syndromes are a group of rare, inherited syndromes characterized by the presence of histopathologically specific or numerous intestinal polyps and an increased risk of cancer. Some polyposis syndromes have been known for decades, but the development in genetic technologies has allowed the identification of new syndromes.. The diagnosis entails surveillance from an early age, but universal guideline on how to manage and surveille these new syndromes are lacking. This paper represents a condensed version of the recent guideline (2020) from a working group appointed by the Danish Society of Medical Genetics and the Danish Society of Surgery on recommendations for the surveillance of patients with hereditary polyposis syndromes, including rare polyposis syndromes.

**Supplementary Information:**

The online version contains supplementary material available at 10.1186/s13053-021-00197-8.

## Background

**Fig. 1:**
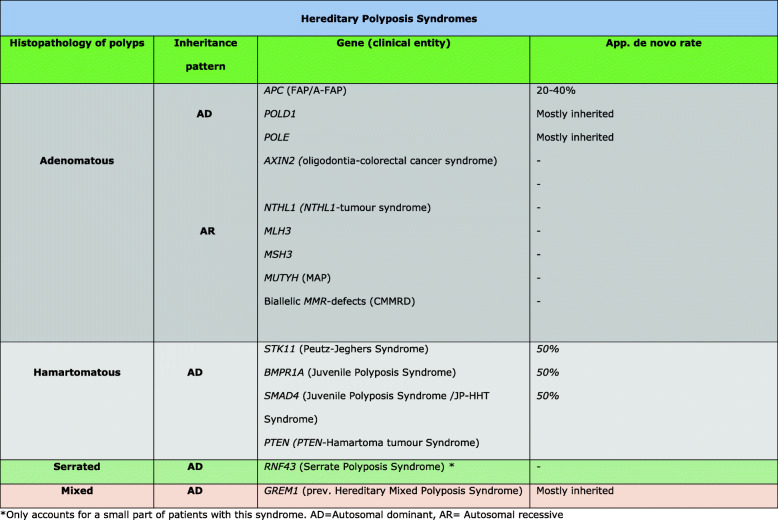
Classification of Hereditary Polyposis Syndromes. *Only accounts for a small part of patients with this syndrome. AD = Autosomal dominant, AR = Autosomal recessive

Hereditary Polyposis Syndromes (HPSs) are a group of rare, inherited syndromes characterized by the presence of histopathologically specific or numerous intestinal polyps and sometimes extra-intestinal manifestations. HPSs is associated with an increased risk of cancer in and outside the gastrointestinal (GI)- tract -tract and timely diagnosis is important in order to offer specific organ-targeted surveillance programs with the purpose of reducing morbidity and mortality. The classification of HPS has traditionally been based on the histopathology of the removed polyps as presented in Fig. 1.

Some HPSs have been known for decades, but the possibility of sequencing many genes in a very short time (Next Generation Sequencing (NGS)), has revealed several genes now known to be associated with HPSs, and genetic testing is therefore a part of the diagnostic pipeline for patients with (or suspected of having) a HPS. Both autosomal dominant and autosomal recessive inheritance is seen.

### Genetics diagnostics and genetic counselling

Genetic testing includes gene-panel screening using NGS with genes known to be related to polyposis syndromes. As of 2020, the panel should include the genes listed in Fig. 1. The finding of causative monoallelic (autosomal dominant) or biallelic (autosomal recessive) germline pathogenic variations (PVs) is crucial in order to make an accurate diagnosis which in turn is the prerequisite for tailoring the optimal surveillance program for each patient. Additionally, detecting a genetic cause also makes prenatal diagnosis, including preimplantation diagnosis (PGT), possible in some cases. Somatic mosaicism should be considered in patients with a clinically convincing HPS, where standard genetic analyses of blood does not identify the cause.

### How to manage HPS?

**Fig. 2:**
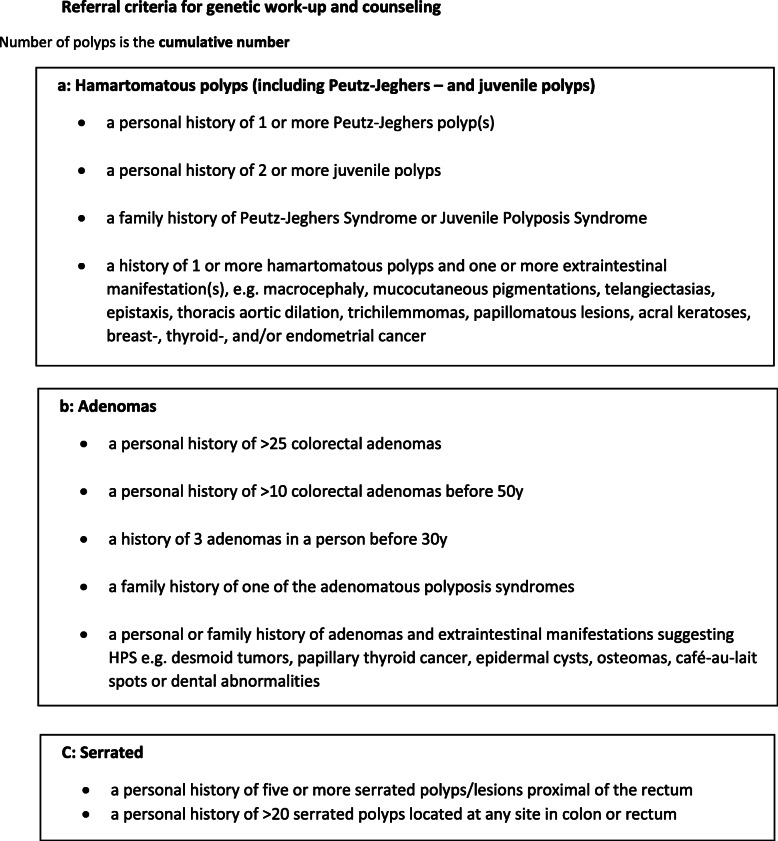
Referral criteria for genetic work-up and counseling. Number of polyps is the cumulative number

There is a high demand for guidelines addressing questions like: how many and what types of polyps should cause concern? When should a patient be referred for genetic counselling? How should we manage patients and their families when detecting (or not detecting) a PV in an HPS gene? In order to address these questions, the Danish Society of Medical Genetics and the Danish Society of Surgery appointed a group of experts in the field in 2017. This paper is a summarized version of this work and guidelines, approved by the two societies in 2020. The guideline points out referral criteria for genetic work-up and counselling (Fig. 2) and suggests surveillance programs for HPS-patients with or without a known genetic etiology. *APC*-associated polyposis and *PTEN*-hamartoma-tumor syndrome are not included in the work. The working group agreed on general recommendations (Suppl. Table 1) and more specific surveillance for each HPS (Suppl. Table 2).

## General considerations of surveillance and prophylactic GI-operations

Endoscopic investigations are the core of surveillance*.* There is no evidence for recommending prophylactic intestinal resections in any of the HPSs, which are described here, but some patients may have a massive polyp burden in part(s) of the GI-tract, making endoscopic surveillance challenging; gastrointestinal resection is indicated in some patients. In the case of colorectal cancer (CRC), a subtotal or total colectomy should be considered, but taking the polyp burden, age and co-morbidity into consideration. If (large) polyps causes complications such as invagination and/or bleeding, segmental resections with or without peroperative enteroscopy should be performed. After surgery surveillance must be resumed.

### HAMARTOMATOUS polyposis syndromes

#### Peutz-Jeghers syndrome

Peutz-Jeghers syndrome (PJS) is characterized by the presence of hamartomatous Peutz-Jeghers polyps in the GI-tract and mucocutaneous pigmentations (MPs) especially on the lips and buccal mucosa. MPs typically presents in childhood and tend to fade after puberty. The polyps are mainly found in the small intestines and 50–75% of patients experience GI symptoms before 20 years of age, with invagination as the most common complication [1]. PJS is inherited in an autosomal dominant manner and *STK11* is the only gene known to be associated with the condition . An age dependent increased risk of cancer in the GI-tract as well as various extra-intestinal cancers are well documented (Table 1) [9]. Surveillance is comprehensive and should start in childhood (see Supp Table 2) [10]*.*

**Table 1:** Estimated cancer risk

Syndrome	Site of cancer	Cumulative lifetime risk or frequency among carriers	Age of debut
**Peutz-Jeghers Syndrome** [2]	Colon/rectum	39%	42-46y
	Stomach	29%	30-40y
	Small bowel	13%	37-42y
	Breast	32–54%	37-59y
	Ovarian	21%	28y
	Cervix (adenoma malignum)	10%	34-40y
	Uterus	9%	43y
	Pancreas	11–38%	41–52y
	Testicular (sertoli cell tumour)	9%	6-9y
	Lung	7–17%	47y
**Juvenile Polyposis Syndrome**	Colon/rectum	38% [3]	36.0 (48) (median)
	Gastric	21% [2]	44.0 (48)
***POLE*-associated polyposis **[4]	Colon/rectum	28% (M), 21%(F) For p.Leu424VAL: 97% (M), 92% (F)	50.2 (49) (mean)
	Uterus	?	
	Ovaries	?	
	Pancreas	?	
	Malignant melanoma	?	
***POLD1*-associated polyposis** [4]	Colon/rectum	90% (M), 82% (F)	39.7 (49) (median)
	Uterus	?	?
	Breast cancer	?	?
	Ovarian	?	?
	Lymphoma	?	?
	Bladder	?	?
***AXIN2*-associated polyposis**	Colon/rectum	13/35 individuals	36–80+
***MUTYH*- associated polyposis** [5]	Colon/rectum	80–90%	48.0 (median)
	Duodenum	4%	61.0
	Ovaries	6–14%	51.0
	Bladder	6–8% (females), 6–25% males)	61.0
	Melanoma	?	?
	Breast	?	?
	Uterus	3%	51.0
***NTHL1-*associated polyposis** [6]	Colon/rectum	16/29 individuals	61.0 median, (33-73y)
	Breast	9/15 female individuals	48.5 (38-63y)
	Uterus	5/15 Uterus (precancerous and cancerous)	57.0 (6–74 y).
	Duodenum	?	
**CMMRD** [7]	Colon/rectum	59/146 individuals	8-48y
	Duodenum	18/149 individuals	11-42y
	Hematologic malignancies	?	¿
	brain tumors		¿
***GREM1*-associated mixed polyposis**	CRC	?	?
**Serrated Polyposis Syndrome **[8]	CRC	15–35%.	53.9 (median)

**Table 2:** Diagnostic criteria

Syndrome	Diagnostic criteria
**Peutz-Jeghers Syndrome**	(1) Two or more histologically confirmed PJS-type hamartomatous polyps, *or* (2) Any number of PJS-type polyps detected in an individual, who has a family history of PJS in (a) close relative(s) *or* (3) Characteristic mucocutaneous pigmentations in an individual, who has a family history of PJS in a close relative(s) *or* (4) Any number of PJS-type polyps in an individual who also has characteristic mucocutaneous pigmentations
**Juvenile Polyposis Syndrome**	(1) More than five juvenile polyps in the colorectum *or* (2) Multiple juvenile polyps throughout the GI-tract *or* (3) Any number of juvenile polyps and a family history of JPS
***POLE*-associated polyposis**	Heterozygosity for a pathogenic missense variant in the exonuclease domain (exon 9–14) of *POLE,* especially p.Leu424Val (Biallelic truncating/splice variants is associated with IMAGe syndrome (MIM: 614732)) and FILS syndrome (MIM: 615139))
***POLD1*-associated polyposis**	Heterozygosity for a pathogenic missense variant in the exonuclease domain (exons 6–12) of *POLD1*
***AXIN2*-associated polyposis**	Heterozygosity for a pathogenic variant in *AXIN2*, especially in exon 8
***MUTYH*-** ***NTHL1-MSH3*- or** ***MLH3*-associated polyposis**	Biallelic pathogenic variants in the relevant gene (*MUTYH, NTHL1, MSH3, MLH3,)*
**Constitutional mismatch repair deficiency syndrome**	Biallelic pathogenic variants in an *MMR* gene (*MLH1, MSH2, MSH6*, and *PMS2, m*ainly *PMS2*. Biallelic carriers of highly penetrant variants are not viable and will die before birth
***GREM1*-associated mixed polyposis**	Heterozygosity for a duplication upstream of *GREM1*
**Serrated Polyposis Syndrome**	(1) At least five serrated lesions/polyps proximal to the rectum, all being 5 or more mm in size with two or more being at least 10 mm in size. (2) More than 20 serrated lesions/polyps of any size distributed throughout the large bowel, with at least five being proximal to the rectum.
**Polyposis with unknown etiology**	(1) At least one family member has had from 20 to 30 (dependent on age) to 99* colorectal polyps, and (2) screening of the relevant genes has not detected a pathogenic variant that can explain the family history, and (3) no other etiology to the gastrointestinal polyps/cancers observed in the family (e.g. genetic syndromes, inflammatory bowel disease etc.) is likely Furthermore, a family history with a significant occurrence of colorectal adenomas/polyps in two or more relatives can indicate a clinically significant predisposition. *If the patient or a relative has had over 100 polyps, the guidelines for FAP should be used

##### Management of patients with a solitary Peutz-Jeghers-polyp or isolated MPs

Patients with a solitary Peutz-Jeghers polyp should be referred to a clinical genetics department for *STK11* analysis. Endoscopy with gastroscopy, colonoscopy and video capsule enteroscopy could be performed in order to rule out PJS. If both genetic and endoscopic work-up is negative, PJS is unlikely and the patient (and family members) should not be subjected to further investigations or follow-up. Isolated MPs suggestive of PJS should be managed as described by *Latchford* et al. [11]

#### Juvenile polyposis syndrome

Juvenile Polyposis Syndrome (JPS) is characterized by the presence of few to over a 100 hamartomatous juvenile polyps in the GI-tract, mostly in the large intestine and stomach. A subgroup of patients with JPS and a PV in *SMAD4* may have symptoms of hereditary hemorrhagic telangiectasia (HHT) as well as an increased risk of aortic aneurisms [12]. The phenotypic spectrum is broad and there is significant intra – and interfamilial variability in expressivity. JPS is inherited in an autosomal dominant manner. The distinction between patients – especially children – with solitary or few juvenile polyps from juvenile polyposis can be difficult, but for patients with only one juvenile polyp the risk for having JPS is low [13]**.** The risk of CRC and gastric cancer is increased with the risk of gastric polyposis, gastric cancer being highest in *SMAD4* carriers [14].

The clinical approach may vary depending on the clinical picture. For *SMAD4* carriers surveillance for HHT should start at 12 years, while the starting point for screening for aortopathy is less clear.

### Autosomal dominant adenomatous polyposis syndromes

#### *POLE*-associated polyposis

PVs in the exonuclease domains *of POLE* (exon 9–14) were described in adult patients with colonic polyps and/or early-onset CRC in 2013 [15, 16]. Since then, additional cases have been reported [17, 18]. Still, data regarding the phenotypic characteristics, penetrance and estimation of cancer risk are limited. Café-au-lait pigmentations may be part of the phenotypic spectrum and are important to discover, as this manifestation can be suggestive of a more aggressive phenotype.

Several PVs have been reported with c.1270C > G, p.Leu424Val, (NM_006231.3) as the most frequent. Other pathogenic missense variants seem to be associated with a more severe phenotype with cancer including medulloblastoma) and CRC [19, 20]. There is an increased risk of CRC, and a high frequency of extraintestinal cancer has been reported. There is no evidence that truncating, loss-of-function variants cause *POLE*-associated polyposis.

The genotype should guide surveillance strategies: p.Leu424Val results in a more Lynch-like phenotype with adult-onset cancer, while other pathogenic missense variants (often de novo) result in a more severe phenotype including childhood cancer and skin pigmentations (see Suppl. Table 2). For carriers of other pathogenic missense variants, surveillance programs should be tailored case by case, but in general beginning in childhood.

#### *POLD1*-associated polyposis

PVs in the exonuclease domains of *POLD1* (exon 6–12) were first described in patients with multiple adenomas and CRCs [21, 22]. Knowledge about phenotype, penetrance and risk of extracolonic cancers is limited, but polyposis and cancer develops in adulthood.

As with POLE, PVs are missense variants and there is no evidence that truncating, loss-of-functions variants cause polyposis and/or cancer.

#### *AXIN2*-associated polyposis

*AXIN2*-associated polyposis (also referred to as oligodontia-colorectal cancer syndrome) is a rare autosomal dominant syndrome, characterized by adenomatous colonic polyps varying in number from 0 to > 100. In most families, affected patients also have oligodontia and/or other aspects of ectodermal dysplasia. There is an increased risk of CRC, which is most often diagnosed in adulthood. Other variants in the gene seem to cause oligodontia/ectodermal dysplasia only [21, 22].

### Autosomal recessive adenomatous polyposis syndromes

#### *MUTYH*- associated polyposis

*MUTYH-*associated polyposis (MAP) is characterized by multiple colorectal adenomas. The phenotypic spectrum is wide, and the number of polyps varies from few to over a 100. Approximately 2% of patients with MAP develop CRC without polyps. The lifetime risk of CRC in patients with MAP is well-documented and high (43–100%). Duodenal adenomas and gastric polyps are also found in a significant part of patients. MAP is caused by biallelic PVs in *MUTYH* thus the inheritance pattern is autosomal recessive. The carrier frequency in the Northern European population is estimated to be 1–2%. Two-point mutations, p.Y179C and p.G396D (NM_001128425), account for approximately. 85% of all PVs in individuals with Northern European ancestry. A high carrier frequency is also found in the Northern African population as well as in the non-Ashkenazi Jewish population.

##### Risk of cancer in heterozygotes with pathogenic MUTYH variants

The risk for colorectal adenomas in monoallelic carriers of pathogenic *MUTYH*-variants has been investigated in a prospective study from 2019 [23] which found no evidence of an adenomatous polyposis phenotype in monoallelic carriers. Carriers of a PV who have a first-degree relative with MAP have up to a 5-fold increased risk of CRC [24], while carriers in general have an over 3-fold increased risk. It is debated whether the finding of one PV in an individual should result in genetic testing of relatives (cascade-testing) as recommendations are contradictive [2, 25]. It is recommended that siblings of a patient with MAP are tested for the PV(s) in the family. Spouses of patients with MAP and spouses of patients, who are heterozygous carriers of a PV should be offered genetic screening of *MUTYH.*

#### *NTHL1-*associated polyposis

*NTHL1-*associated polyposis (or *NTHL1*-tumour syndrome) was described for the first time by *Weren* et al. in 2015 in patients with adenomatous polyposis in the lower GI-tract [26]. As of January 2020, reports of 34 patients with *NTHL1*-associated polyposis have been published [6, 26–32]. Development of *NTHL1*-associated polyposis is caused by biallelic PVs in *NTHL1* and the inheritance pattern is autosomal recessive. Most patients are homozygous for the recurrent PV, *NTHL1,* c.268C > T, p.Gln90* (NM_002528).

There is a high frequency of CRC in the published cases, but also of breast- and duodenal cancer suggesting a broader cancer predisposition syndrome [32–39]. Thus, the phenotypic spectrum of this syndrome is still emerging.

#### Constitutional mismatch repair deficiency syndrome

Constitutional mismatch repair deficiency syndrome (CMMRD) is a distinct childhood cancer predisposition syndrome characterized by an increased risk of a broad spectrum of malignancies, and GI-polyposis in both the upper and lower GI-tract. Often café-au-lait spots and other findings that mimic neurofibromatosis type 1 are detected. The patients carry biallelic PVs in the MMR genes (*MLH1, MSH2, MSH6* and *PMS2*) and thus the inheritance pattern is autosomal recessive. More than half of the patients known with CMMRD have bi-allelic PVs in *PMS2* [33]*.* Recommendations for GI-surveillance are listed in Suppl.Table 2. Suggested surveillance for other malignancies is described by the European Consortium of CMMRD [34].

#### *MSH3*- and *MLH3*-associated polyposis

*MSH3:* As of January 2020, a total of four individuals from two families have been reported with biallelic PVs in *MSH3* [35]. The inheritance pattern is autosomal recessive and the associated phenotype is characterized by the presence of colorectal adenomatous polyposis. Polyposis was accompanied by benign and malignant lesions in the GI- tract and extracolonic manifestations such as duodenal adenomas, thyroid adenomas, gastric cancer and astrocytoma.

*MLH3: Olkinuora* et al. [36] reported five patients from four families to be homozygous for PVs in *MLH3,*. The patients had 50–200 adenomatous polyps (age range 48–52 years). One of three female patients had breast cancer at age 52, and the male patient had CRC at age 48.

### Other polyposis syndromes

#### *GREM1*-associated mixed polyposis

*GREM1*-associated Mixed Polyposis (previously Hereditary Mixed Polyposis syndrome) is an extremely rare condition with an unknown incidence. The condition was first described by *Whitelaw* et al. in 1997 [37] in an Ashkenazi Jewish family with mixed GI-polyposis and CRC. A genetic cause was reported in 2012 by *Jaeger* et al, who detected a 40 kb duplication upstream of *GREM1* [38]. Since then other duplications have been reported [21, 39, 40]. The mode of inheritance is autosomal dominant and the condition is caused by upstream *GREM1* duplications [40]. The histopathology of the polyps is variable and includes atypical juvenile polyps and/or hyperplastic polyps as well as adenomas and serrated adenomas, and there is a phenotypic overlap with other syndromes, although the phenotypic description is limited. CRC occurs with an increased frequency in adulthood [41].

#### Serrated polyposis syndrome

Serrated Polyposis Syndrome (SPS) (previously named Hyperplastic Polyposis Syndrome) Is a condition characterized by numerous serrated polyps in the colon. Although the prevalence is unknown, the syndrome is probably more common than anticipated. In fecal occult blood test-based screening cohorts 1:111–1:238 individuals were diagnosed [42, 43].

SPS is commonly grouped with the HPSs but does not appear to be inherited in a simple Mendelian fashion. Some studies link PVs in *RNF43* to SPS; however, studies of larger cohorts suggest that *RNF43* only explains a small proportion of cases [44, 45]. Individuals with SPS have an increased risk of CRC, and relatives have a recognized substantial risk of developing CRC, but the risk is not well defined [46].

#### Polyposis with unknown etiology

In some patients with a significant number of adenomas in the L-GI-tract, both with and without a family history of polyposis, the etiology is not identified by gene analysis. These patients/families may have the diagnosis of “polyposis of unknown etiology” although there is no clear definition of the term “polyposis”. The definition seen in Table 2 can be used as guidance.

Few publications have focused on this group of patients, and these are likely influenced by selection bias. Cancer occurrence has been reported in relatives, both in the colon and in the U-GI tract*,* but the inclusion criteria in these studies differ from those listed in Table 2 [47–49]. The National Comprehensive Cancer Network (NCCN) suggests surveillance/management guided by the phenotype of the patient and by the family history [2].

## Conclusion

In recent years, HPSs have been identified due to the development in genetic technologies. Patients with these syndromes should be offered surveillance in order to reduce mortality and morbidity, and genetic analysis is crucial in the diagnostic pipeline. Long-term follow-up studies are needed in order to obtain evidence but are complicated by the small number of patients, lack of population-based data and risk of ascertainment bias. The guidelines presented will have to undergo revision as knowledge increases and new polyposis syndromes are identified.

## Supplementary Information

**Additional file 1: Supplementary Table 1**: General recommendations for management of the Hereditary Polyposis Syndromes.

**Additional file 2: Supplementary Table 2**: Surveillance strategies for each Hereditary Polyposis Syndrome.

## Data Availability

Not applicable.

## Abbreviations

CMMRD: Congenital mismatch repair deficiency
CRC: Colorectal cancer
FAP: Familial adenomatous polyposis
FDR: First-degree relative
HHT: Hereditary hemorrhagic telangiectasia
HPS: Hereditary polyposis syndrome
MP: Mucocutaneous pigmentations
JPS: Juvenile polyposis syndrome
L-GI: Lower gastrointestinal tract
MAP: *MUTYH-*associated polyposis
PJS: Peutz-Jeghers syndrome
PV: Pathogenic variant
U-GI: Upper gastrointestinal tract
SPS: Serrated Polyposis Syndrome
VCE: Videocapsule endoscopy
