# Supplementary material for: Danish guidelines for management of non-APC-associated hereditary polyposis syndromes
Source: Hered Cancer Clin Pract. 2021 Oct 7;19:41. doi: 10.1186/s13053-021-00197-8 (PMC8499431; doi:10.1186/s13053-021-00197-8)
Supplement: Supplementary file 1 — Additional file 1: Supplementary Table 1: General recommendations for management of the Hereditary Polyposis Syndromes. [file 13053_2021_197_MOESM1_ESM.docx]

**Supplementary Table 1: General recommendations for management of the Hereditary Polyposis Syndromes**

| A | All patients diagnosed with a HPS should be registered in a national register to promote research as well as equal and sufficient health care irrespectively of where the patient lives (In Denmark registration is performed in the Danish HNPCC register) |
| --- | --- |
| B | Patients suspected of, or diagnosed with a HPS and/or having a family history with HPS, should be referred for genetic counseling and testing |
| C | Genetic testing in a patient, whose phenotype does not point towards a specific HPS, should include at least the following genes: *APC, AXIN2, POLE, POLD1, MUTYH, NTHL1, MSH2, MLH1, MLH3, MSH3, MSH6, STK11, SMAD4, BMPR1A, PTEN, RNF43, GREM1, MLH2, PMS2* |
| D | Mosaicism should be considered in patients strongly suspected of HPS but where genetic testing is negative. |
| E | When deciding on a surveillance program for HPS patients and/or family members at risk, one should consider both patient- and family histories, including age at diagnosis of polyposis and cancer occurrences (age and site). |
| F | Colectomy or subtotal colectomy as well as gastrectomy should be considered in patients with a polyp burden not manageable by endoscopy. |
