# Supplementary material for: Danish guidelines for management of non-APC-associated hereditary polyposis syndromes
Source: Hered Cancer Clin Pract. 2021 Oct 7;19:41. doi: 10.1186/s13053-021-00197-8 (PMC8499431; doi:10.1186/s13053-021-00197-8)
Supplement: Supplementary file 2 — Additional file 2: Supplementary Table 2: Surveillance strategies for each Hereditary Polyposis Syndrome. [file 13053_2021_197_MOESM2_ESM.docx]

**Supplementary Table 2:** Surveillance strategies for each Hereditary Polyposis Syndrome

| **Syndrome/Site** | **Age for surveillance (years)** | **Surveillance interval (years)** | **Surveillance procedures** | **Comments** |
| --- | --- | --- | --- | --- |
| **Peutz-Jeghers Syndrome** |  |  |  |  |
| U-GI | 8- | 3 (from 50y: 2y) | Esophagogastroduodenoscopy with polypectomy of polyps> 5 mm | Surveillance should begin earlier if symptoms occur.  If no polyps are found at age 8, repeat at 18y |
| Small intestines | 8- | 3 (from 50y: 2y) | VCE with polypectomy of polyps > 20 mm (preferably by device-assisted enteroscopy) | Surveillance should begin earlier if symptoms occur. |
| Colon | 8- | 3 (from 50y: 2y) | Colonoscopy with polypectomy > 5 mm. | Surveillance should begin earlier if symptoms occur.  If no polyps are found at age 8, repeat at 18y |
| Pancreas | 40- | 1 | MR-CP/EUS | Or 10 years before the earliest case of pancreatic cancer in the family |
| Ovaries/Cervix | 25- | 1 | Vaginal UL-sound, CA125 and SMEAR | Patients should be advised to react on increased vaginal discharge.  PBSO could be considered after reproduction |
| Breast (female) | 30-70 | 1 | Clinical breast exam, mammography+ MRI | Prophylactic mastectomy could be considered  After 70y. mammography every second year. Mammography without MR can be sufficient in some after a radiologist evaluation. See also ^2)^ |
| Testes | 0 (birth)-12 | 1 | Clinical exam and UL if abnormalities are suspected. |  |
| *FDRs of STK11-negative PJS patients* | 8- | See comments | Dermatological evaluation for mucocutaneous pigmentations | If no mucocutaneous pigmentations are seen, gastroscopy, colonoscopy and VCE should be performed at age 18y. If no hamartomatous polyps are detected, there is no need for follow-up. If polyps are detected the PJS surveillance program should be followed. |
| **Syndrome/Site** | **Age for surveillance (years)** | **Surveillance interval (years)** | **Surveillance procedures** | **Comments** |
| **Juvenile Polyposis Syndrome** |  |  |  |  |
| U-GI | 12-75 | 3 | Esophagogastroduodenoscopy with polypectomy and polyps > 5 mm | Surveillance should begin earlier if symptoms occur. |
| Colon | 12-75 | 3 | Colonoscopy with polypectomy of polyps > 5 mm | Surveillance should begin earlier if symptoms occur. |
| *JP-HHT SMAD4- carriers* |  |  |  |  |
| GI | As under Juvenile Polyposis syndrome |  | - |  |
| Aortopathy/HHT | Refer to the Danish HHT-center/specialist |  | - |  |
| *FDRs to a patient with JPS without a PV* |  |  |  |  |
| U-GI | Single investigation between 15-18y |  | Esophagogastroduodenoscopy with polypectomy of all polyps | If no hamartomatous polyps are detected no need for follow-up. If polyps are detected follow-up as for PV carriers |
| L-GI | Single investigation 15-18y |  | Colonoscopy with polypectomy of all polyps | If no hamartomatous polyps are detected no need for follow-up. If polyps are detected follow-up as for PV carriers |
| *POLE*-associated polyposis c.1270C>G, p.Leu424Val |  |  |  |  |
| U-GI | 20- | Spiegelman criteria should guide screening intervals | Esophagogastroduodenoscopy preferably with side-viewing instruments. Polypectomy, EMR, APC, or papillectomy of duodenal polyps > 10 mm. Duodenal polyps < 10 mm should be biopsied. Suspicious lesions in the stomach should be biopsied or removed, but classic fundic gland hyperplasia of the fundus can be left untouched. |  |
| Colon | 20-70 | 2 | Colonoscopy with polypectomy of polyps > 5 mm |  |
| Skin | 25- | 1 | Dermatological evaluation |  |
| Ovaries/uterus | 35- | 1 | Vaginal UL-sound and CA125 |  |
| *Carriers of other missense PVs* |  |  |  | Surveillance should be tailored taking the family history in consideration; but in general surveillance should start in childhood. |
| Syndrome/Site | **Age for surveillance (years)** | **Surveillance interval (years)** | **Surveillance procedures** | **Comments** |
| *POLD1*-associated polyposis |  |  |  |  |
| U-GI | 20-70 | Spiegelman criteria should guide screening intervals | Esophagogastroduodenoscopy preferably with side-viewing instruments. Polypectomy, EMR, APC or papillectomy of duodenal polyps > 10 mm. Duodenal polyps < 10 mm should be biopsied. Suspicious lesions in the stomach should be biopsied or removed, but classic fundic gland hyperplasia of the fundus can be left untouched. |  |
| Colon | 20-70 | 2 | Colonoscopy with polypectomy of polyps > 5 mm |  |
| Ovaries/Uterus | 35- | 1 | Vaginal UL-sound/CA125 |  |
| ***AXIN2*-related polyposis** |  |  |  |  |
| Colon | 20-75 | 2 | Colonoscopy with polypectomy of polyps > 5 mm | Surveillance can start earlier if family history suggests polyps occurring prior to 20y |
| ***MUTYH*-associated polyposis (biallelic carriers)** |  |  |  |  |
| U-GI | 35-75 | Spiegelman criteria should guide screening intervals | Esophagogastroduodenoscopy preferably with side-viewing instruments. Polypectomy, EMR, APC or papillectomy of duodenal polyps > 10 mm. Duodenal polyps < 10 mm should be biopsied. Suspicious lesions in the stomach should be biopsied or removed, but classic fundic gland hyperplasia of the fundus can be left untouched. |  |
| Colon | 20-75 | 1 | Colonoscopy with polypectomy of polyps > 5 mm |  |
| ***MUTYH*-associated risk (monoallelic carriers) If CRC in an FDR** |  |  |  |  |
| Colon | 50-75 | 5 | Colonoscopy with polypectomy |  |
| ***NTHL1*-associated polyposis** |  |  |  |  |
| U-GI | 40-75 | 2-3 | Polypectomy, EMR, APC or papillectomy of duodenal polyps > 10 mm. Duodenal polyps < 10 mm should be biopsied. Suspicious lesions in the stomach should be biopsied or removed, but classic fundic gland hyperplasia of the fundus can be left untouched. |  |
| **Syndrome/Site** | **Age for surveillance (years)** | **Surveillance interval (years)** | **Surveillance procedures** | **Comments** |
| ***NTHL1*-associated polyposis (continued)** |  |  |  |  |
| Colon | 25-75 | 2-3 | Colonoscopy with polypectomy of polyps > 5 mm | If adenomas are found, colonoscopy every 1-2 years. |
| Breast | 40-49 | 1 | Clinical mammography | Hereafter national screening program every second year |
| Uterus | 40-75 | 2 | Vaginal UL |  |
| **CMMRD** |  |  |  |  |
| U-GI | 10- | 1 | Esophagogastroduodenoscopy with polypectomy |  |
| Small intestines | 10- | 1 | VCE |  |
| Colon | 8- | 1 | Colonoscopy with polypectomy |  |
| Other organ systems see Vasen et al. (2014)(1) |  |  |  |  |
| ***MSH3*-and *MLH3*-associated Polyposis** |  |  |  |  |
| U-GI | 40-75 | 2 | Esophagogastroduodenoscopy with polypectomy | If adenomas are found, colonoscopy every year. |
| Colon | 25-75 | 2-3 | Colonoscopy with polypectomy |  |
| ***GREM1*- associated** **Mixed Polyposis (HMPS)** |  |  |  |  |
| Colon | 20-50 | 3 | Colonoscopy with polypectomy |  |
| Colon | 51-70 | 2 | Colonoscopy with polypectomy |  |
| **Serrated Polyposis Syndrome** |  |  |  |  |
| Colon (affected) | From diagnosis | 1-2 | Colonoscopy with polypectomy of polyps > 5 mm |  |
| FDR or *RNF43*-carriers with a FDR with CRC | 40-60 | 5 | Colonoscopy with polypectomy of polyps > 5 mm | or 10 years earlier than the earliest diagnosis of CRC in the family. If FDRs is over 60 y a single colonoscopy is recommended. |
| **Syndrome/Site** | **Age for surveillance (years)** | **Surveillance interval (years)** | **Surveillance procedures** | **Comments** |
| **Polyposis without known etiology** |  |  |  |  |
| Individuals with between 20-30 and 99 colorectal adenomas | From time of diagnosis to 75 | 3 | Colonoscopy with polypectomy | If over 100 colorectal adenomas are detected, recommendations for familial adenomatous polyposis (FAP) should be used. |
| FDR to affected relative | 40- (see comment) | 3 | Colonoscopy with polypectomy | Surveillance should start  at the age of the youngest relative with polyposis, or  5 years before the youngest relative with CRC, or  from 40 y, whichever is earliest. |

**Abbreviations:** APC = argon plasma koagulation, EMR= endoscopic mucosa resection, FDR= first-degree relative, JPS= Juvenile Polyposis Syndrome, PV=pathogenic variant, VCE= video capsule endoscopy

1. Vasen HF, Ghorbanoghli Z, Bourdeaut F, Cabaret O, Caron O, Duval A, et al. Guidelines for surveillance of individuals with constitutional mismatch repair-deficiency proposed by the European Consortium "Care for CMMR-D" (C4CMMR-D). Journal of medical genetics. 2014;51:283-93.

2. Danish Society of Medical Genetics guideline for hereditary breast cancer: https://dsmg.dk/wp-content/uploads/2019/11/Henvisningskriterier-version-1-december-2019_red.pdf
